# Soluble biglycan: a potential mediator of cartilage degradation in osteoarthritis

**DOI:** 10.1186/s13075-015-0902-0

**Published:** 2015-12-24

**Authors:** Goncalo Barreto, Antti Soininen, Pekka Ylinen, Jerker Sandelin, Yrjö T. Konttinen, Dan C. Nordström, Kari K. Eklund

**Affiliations:** Department of Internal Medicine and Rehabilitation, University of Helsinki and Helsinki University (Central) Hospital, Biomedicum 1, PO Box 63, FIN-00290 Helsinki, Finland; Department of Rheumatology, University of Helsinki and Helsinki University (Central) Hospital, Helsinki, Finland; ORTON Orthopaedic Hospital, Helsinki, Finland

**Keywords:** Biglycan, Decorin, Osteoarthritis, Toll-like receptors, Chondrocytes, Cartilage, Innate immune response

## Abstract

**Background:**

Soluble biglycan (sBGN) and soluble decorin (sDCN), are two closely related essential components of extracellular matrix which both have been shown to possess proinflammatory properties. We studied whether sBGN or sDCN were present in synovial fluid (SF) of osteoarthritis (OA) or rheumatoid arthritis (RA) patients and studied sBGN or sDCN potential role in the degradation of OA cartilage.

**Methods:**

SF obtained from meniscus tear, OA, and RA patients were analysed for sBGN and sDCN using enzyme-linked immunosorbent assays. OA chondrocytes and cartilage explants were stimulated for 48 h with 5 μg/ml sBGN or 1 μg/ml lipopolysaccharide. Messenger RNA (mRNA) levels of Toll-like receptors (TLRs), proteinases and cartilage matrix molecules were determined using quantitative real-time polymerase chain reaction. Protein levels of matrix metalloproteinases (MMPs) and cytokines were measured using Luminex xMap technology. Production of nitric oxide (NO), release of proteoglycans and soluble collagen were measured from conditioned culture media using biochemical assays. OA cartilage explant proteoglycans were stained for Safranin O and quantified using image analysis. TLR4 activation by sBGN and sDCN was studied in engineered HEK-293 cells with TLR4 signalling genes inserted together with a reporter gene.

**Results:**

sBGN was found in meniscus tear SF (14 ± 2 ng/ml), OA SF (582 ± 307 ng/ml) and RA SF (1191 ± 482 ng/ml). Low levels of sDCN could also be detected in SF of meniscus tear (51 ± 4) ng/ml, OA (52 ± 3 ng/ml), and RA (49 ± 4 ng/ml). Stimulation of chondrocytes with sBGN increased significantly the mRNA and protein expression of catabolic MMPs, including MMP1, MMP9 and MMP13, and of inflammatory cytokines interleukin (IL)-6 and IL-8, whereas the expression of anabolic markers aggrecan and collagen type II was decreased. sBGN induced release of proteoglycans, collagen and NO from chondrocytes and cartilage explants. The catabolic response in explants was dependent of OA cartilage degradation stage. The mechanism of action of sBGN was mainly mediated through the TLR4-nuclear factor-κB pathway.

**Conclusions:**

High levels of sBGN was found in advanced OA and RA SF. sBGN activates chondrocytes mainly via TLR4, which results in net loss of cartilage. Thus, sBGN can be a mediator of OA cartilage degradation and also a potential biomarker for arthritis.

## Background

Osteoarthritis (OA) is among the top five leading causes of disability worldwide [1]. To date, no effective long-term disease-modifying treatment for OA is available. At the root of the OA burden lies poor comprehension of the molecular pathophysiology of pre-symptomatic and clinically symptomatic OA.

Cartilage degeneration, a hallmark of OA, has its onset in wear, tear and mechanical injuries of the joint cartilage. Although trauma is perhaps the first causal event in OA, the host inflammatory response plays an important role in the pathogenesis OA and inflammation is believed to be the major driver to symptomatic OA [2, 3].

Cells of the cartilage, known as chondrocytes, maintain cartilage tissue homeostasis. They control the structural assembly of extracellular matrix (ECM) and regulate destructive, remodelling and reparative processes [4]. In OA, cartilage-resident chondrocytes produce proteinases such as aggrecanases (ADAMTS-4, ADAMTS-5), matrix metalloproteinases (e.g., MMP-13 and MMP-9) and cathepsin K, and the anabolic synthesis of structural molecular components aggrecan and collagen type II (Col-II) are compromised [5]. Such a metabolic imbalance results in a failure of cartilage homeostasis and pathological cartilage destruction as well as eventual loss of cartilage.

ECM molecules have been thought to function as purely structural matrix-bound components, but evidence is emerging that they can also function as soluble ligands for pattern-recognising danger signalling receptors, mainly for Toll-like receptors (TLRs) [6–8]. Several of the ECM molecules found in OA joint and synovial fluid (SF) have been shown to trigger catabolic responses in TLR-equipped OA chondrocytes [9–11]. Biglycan (BGN) and decorin (DCN) are two small, closely related structural proteoglycans with leucine-rich repeats (small leucine-rich proteoglycan, SLRP) [12, 13]. The soluble forms of BGN (sBGN) or (sDCN) released from the cartilage matrix as a result of tissue injury could potentially function as an endogenous danger signal [14]. For example, sBGN, the size of which is approximately 95 kDa, has been shown trigger TLR4/TLR2 signalling in human aortic valve and in acute ischemic kidney, as well as to activate the NLRP3 inflammasome in macrophages [15–17]. sDCN has been shown to activate TLR4/TLR2 signalling in macrophages [18].

We hypothesised that BGN is a prototypic example of a major essential cartilage ECM component. Upon release from stressed cartilage, it might activate TLR-mediated catabolic signalling pathways [19, 20]. In fact, several studies have shown that de novo BGN and DCN synthesis is increased in OA, that BGN and DCN fragments are produced and released by ADAMTSs and MMPs, and that OA SF also contains BGN and DCN autoantibodies [21–25]. However, to our knowledge, no studies have addressed the concentration of intact sBGN and sDCN in OA or rheumatoid arthritis (RA) SF or included examination of the eventual direct role of these proteoglycans in chondrocyte-mediated cartilage degradation in OA.

Our aim was to study whether sBGN and sDCN can be found in SF from arthritic joints and whether sBGN and sDCN can contribute to cartilage degradation by activating the TLR-mediated catabolic signalling in primary OA chondrocytes and OA tissue explants.

## Methods

### Tissue acquisition, cartilage explant culture and primary chondrocyte culture

Patient recruitment and participation and sample collection were approved by the Helsinki and Uusimaa Hospital District ethics committee (Dnr_59/13/03/02/2013) and hospital board of directors (9310/407), and signed informed consent was obtained from the patients. Samples were collected from patients with OA who underwent total knee arthroplasty (TKA) (N = 12). Half of the patients were women. The mean age of the patients was 63.5 years (range 53–73). Tibial plateau containing cartilage and subchondral bone was cut off to provide space for the tibial component of the TKA implant. Tibial plateau samples were collected into sterile sample containers containing cold phosphate-buffered saline solution (PBS) and processed within 2 h.

Before tissue samples were harvested, the macroscopic appearance of the visible pathological changes in different sampling areas was recorded according to the arthroscopic grading scale of Société Française d’Arthroscopie [26]. Explant samples were collected from two different types of areas: grade I OA samples with no macroscopically visible lesions (which represents early OA cartilage degradation stages) or grade IV samples with full-thickness osteochondral lesions [27]. This grading of the samples makes possible a stratified analysis based on the degree of OA cartilage, from very mild to severe. Cylindrical full-thickness cartilage explants were obtained from graded tibial plateau areas using a 4-mm circular punch biopsy blade (Kai Medical, Oyana, Japan). Explants were cultured in Gibco Dulbecco’s modified Eagle’s medium (DMEM)/F-12 (Life Technologies, Carlsbad, CA, USA) supplemented with 10 % foetal calf serum (FCS) for 24 h before stimulation.

When cartilage explants were not isolated, cartilage tissue (with bone cut away) was used for chondrocyte isolation. Chondrocytes form only 1–3 % of the volume of the cartilage, so the whole cartilage was used for chondrocyte isolation to obtain enough cells. This means that the chondrocytes represent a heterogeneous population of OA chondrocytes. This does not allow a stratified analysis. Cartilage specimens were sequentially digested in 2.5 mg/ml pronase and 250 mg/ml collagenase P (Roche, Basel, Switzerland), with a PBS wash in between, for 60 minutes and overnight, respectively, under slow agitation at 37 °C. The resulting cell suspension was filtered through a 70-μm nylon cell strainer, centrifuged, washed twice with PBS and seeded at 1.5 × 10^5^/cm^2^ in Gibco DMEM/F-12 supplemented with 10 % FCS, 100 U/ml penicillin, 100 U/ml streptomycin and 0.25 μg/ml amphotericin B (Life Technologies). Cells were balanced for 3–4 days before stimulation. For cryopreservation, cells were suspended in FCS containing 10 % (vol/vol) dimethyl sulphoxide and frozen in an isopropanol container for 24 h at −80 °C before storage in liquid nitrogen.

### Arthritis group definitions for synovial fluid collection

#### Early osteoarthritis

SF samples were collected from ten patients scheduled for arthroscopic surgery. The main indication for surgery was a suspicion of a meniscal tear. Patients with inflammatory arthritis, severe arthritis, corticosteroid injection within 6 weeks, blood dyscrasias or active malignancy were excluded. The prior use of nonsteroidal anti-inflammatory drugs was not considered an exclusion criterion. The diagnosis of early arthritis was made during arthroscopy and based on the presence of minimal visible chondral lesions.

#### Advanced osteoarthritis

SF samples were collected from ten patients scheduled for elective total knee replacement for management of primary idiopathic OA. The exclusion criteria were similar to those for patients with early OA.

#### Rheumatoid arthritis

SF samples were collected from ten patients with RA diagnosed fulfilling the American College of Rheumatology/European League Against Rheumatism classification criteria for RA [28].

### Synovial fluid sample collection

Patients with early OA, advanced OA and RA were, depending on the schedule of the surgeon, randomly selected for collection of SF samples via needle aspiration before opening of the joint. Blood-contaminated samples were excluded. SF maintained at 4 °C was aliquoted within 2 h into sterile Eppendorf tubes, centrifuged at 1200 *g* for 5 minutes at room temperature to separate solid debris and cells from the fluid phase, snap-frozen in liquid nitrogen and stored at −80 °C. When first thawed, SF was treated with a protease inhibitor cocktail (Roche Diagnostics, Meylan, France).

### Enzyme-linked immunosorbent assay

SF was measured for intact-only sBGN and DCN molecules using a specific sandwich enzyme-linked immunosorbent assay (ELISA) (Uscn Life Science Inc., Hubei, China, and BioVendor Laboratorní medicína, Brno, Czech Republic, respectively) for detection of intact sBGN and sDCN molecules. BGN and DCN fragments are not detected by the immunoassays. Absorbance was measured at 450 nm, as well as 450 nm and 630 nm, for sBGN and sDCN immunoassays, respectively. All measurements were performed in duplicates.

### SEAP NF-κB activity assays

TLR4 activity was measured using a cell-based assay according to the manufacturer’s instructions (InvivoGen, San Diego, CA, USA). HEK-hTLR4 cells express *TLR4* and *MD-2/CD14* co-receptor genes of human origin and contain the secreted embryonic alkaline phosphatase (*SEAP*) reporter gene for monitoring nuclear factor (NF)-κB activation. Upon interaction with the appropriate ligand, TLR4 transduces a signal which results in NF-κB activation and the expression of secreted alkaline phosphatase (AP), which can be detected by using detection medium (QUANTI-Blue, a medium used for the detection and quantification of secreted AP; InvivoGen) and measured with a spectrophotometer.

Briefly HEK-hTLR4 cells were cultured at a density of 2.5 × 10^4^ cells in 96-well plates and maintained in complete DMEM with selective antibiotics, as described in the manufacturer’s instructions (InvivoGen). Cells were then stimulated with 5 μg/ml BGN (Sigma-Aldrich, St. Louis, MO, USA), 8 μg/ml sDCN (R&D Systems, Minneapolis, MN, USA) or 1 μg/ml lipopolysaccharide (LPS) (positive control) for 24 h, and activation of TLR4 and NF-κB signalling was analysed by measuring SEAP from conditioned culture medium samples according to the manufacturer’s instructions (InvivoGen).

### Stimulation of chondrocyte and cartilage explant cultures

Primary chondrocytes cultures from passage 1 were stimulated for 24 or 48 h with 5 μg/ml bovine BGN (high sequence homology and structural conservation with human BGN) obtained from articular cartilage and solubilised according to the manufacturer’s instructions (Sigma-Aldrich) [29]. BGN endotoxin (LPS) contamination was not detected using the limulus amebocyte lysate test. As additional endotoxin controls, 5 μg/ml sBGN was incubated in fresh Gibco DMEM/F-12 containing 50 μg/ml proteinase K (Thermo Scientific, Waltham, MA, USA) or 25 μg/ml polymyxin B (InvivoGen) at 37 °C for 1 h, after which the mixtures were added to the primary chondrocyte cultures. Protein homogeneity was confirmed by sodium dodecyl sulphate–polyacrylamide gel electrophoresis and Coomassie staining (performed by the manufacturer). LPS (Sigma-Aldrich) stimulation at 1 μg/ml was used as a positive TLR2/TLR4 control. In selected experiments, 1 μM CLI-095 (InvivoGen) was added to primary chondrocyte cultures 30 minutes before sBGN or LPS. CLI-095, also known as TAK-242, specifically suppresses TLR4 signalling mediated by the intracellular domain of the receptor. Further receptor-blocking studies were performed with the addition of 20 μg/ml TLR2 (Santa Cruz Biotechnology, Santa Cruz, CA, USA) or 20 μg/ml TLR4 (Santa Cruz Biotechnology) for 1 h before the addition of sBGN at 5 μg/ml.

The optimal concentration for sBGN stimulation was determined in 24- and 48-h pilot experiments using 0.05, 0.5 and 5 μg/ml of sBGN, followed by analysis of *MMP13*, *CTSK* (cathepsin K, cat K), *IL6* (interleukin-6, or IL-6) and *COL2A1* (collagen type II α chain 1, or Col-IIA) gene messenger RNA (mRNA) copy numbers relative to the TATA box-binding protein (*TBP*) housekeeper, by using quantitative real-time polymerase chain reaction (qRT-PCR). Cartilage explant cultures were stimulated using the same conditions.

### Real-time polymerase chain reactions

Total RNA was isolated from cartilage explants by using the RNAqueous® kit (Thermo Scientific) and from primary chondrocytes by using the RNeasy® Mini Kit (Qiagen, Valencia, CA, USA). Relative quantification of the gene levels was performed by comparing the cycle threshold (C_t_) values of the different genes, correcting for glyceraldehyde 3-phosphate dehydrogenase (*GAPDH*) and *TBP* content (∆C_t_) and for non-stimulated conditions (∆∆Ct) and finally expressed as fold changes. Primer sequences are provided in Table 1.

**Table 1:** Primer pairs used for real-time polymerase chain reactions

Gene	Protein name	Primer sequence
*ADAMTS4*	A disintegrin and metalloproteinase with thrombospondin motifs 4	Forward: 5′-AAT CCT GTC AGC TTG GTG GT-3′
		Reverse: 3′-CTT GGA GTT GTC ATG GAG CA-5′
*ADAMTS5*	A disintegrin and metalloproteinase with thrombospondin motifs 5	Forward: 5′-CTT CAC TGT GGC TCA CGA AA-3′
		Reverse: 3′-AAT GTC AGG TTG CAC TGC TG-5′
*MMP13*	Matrix metalloproteinase 13 (MMP-13)	Forward: 5′-CTA TGG TCC AGG AGA TGA AG-3′
		Reverse: 3′-AGA GTC TTG CCT GTA TCC TC-5′
*CTSK*	Cathepsin K	Forward: 5′-ACC CAA CAG GCA AGG CAG CTAA-3′
		Reverse: 3′-GCA ATG CCA CAGG CGT TGT TCT-3′
*MMP9*	Matrix metalloproteinase 9 (MMP-9)	Forward: 5′-TTC TCC AGA AGC AAC TGT CC-3′
		Reverse: 3′-CGG CAA GTC TTC CGA GTA GT-5′
*ACAN*	Aggrecan	Forward: 5′-TGG TGA TGA TCT GGC ACGA-3′
		Reverse: 3′-TCT GCG TTT GTA GGT GGTG-5′
*COL2A1*	Collagen, type II, alpha 1	Forward: 5′-GAG TCA AGG GTG ATC GTG GT-3′
		Forward: 5′-AAG CAC CTT GGT CTC CAG AA-5′
*TBP*	TATA box-binding protein	Forward: 5′-GAA GAA CAA TCC AGA CTA GCA GCA-3′
		Reverse: 3′-CCT TAT AGG GAA CTT CAC ATC ACAG-5′
*GAPDH*	Glyceraldehyde 3-phosphate dehydrogenase	Forward: 5′-AAG GTC ATC CCT GAG CTG AA-3′
		Reverse: 3′-TGC TGT AGC CAA ATT CGT TG-5′

### Protein measurements using Luminex xMAP® technology

Measurement of protein levels was done using xMAP® technology (Luminex, Austin, TX, USA). To determine protein levels of MMPs (MMP-1, MMP-3, MMP-9 and MMP-13) as well as cytokines and chemokines (IL-6, IL-8) in chondrocyte culture supernatant, xMAP® technology on the Bio-Plex 200® system (Bio-Rad Laboratories, Hercules, CA, USA) was used in combination with multiplex MMP/cytokine kits (ProcartaPlex; eBioscience; San Diego, CA, USA). Protein levels were measured in 25 μl of culture medium (diluted 1:2).

### Measurement of nitric oxide

Nitric oxide (NO) was measured from conditioned culture medium samples using a nitrate/nitrite colorimetric assay kit (Cayman Chemical, Ann Arbor, MI, USA). Nitrate was converted to nitrite by adding nitrate reductase and its co-factor, followed by the addition of Griess reagent to develop a deep purple colour. The absorbance was measured at 544 nm using a plate reader (Chameleon; Hidex, Turku, Finland).

### Measurement of soluble sulphated glycosaminoglycans and soluble collagen type II

Soluble sulphated glycosaminoglycan (sGAG) standards, blanks and conditioned culture medium samples were mixed with the sGAG-binding Blyscan Dye Reagent® for 30 minutes, followed by separation of the GAG–dye complex by centrifugation and dissociation of the dye from the pellet (Biocolor Ltd., Carrickfergus, UK). Soluble Col-II was similarly mixed with the collagen-binding Sircol Dye Reagent® (Biocolor Ltd.) for 30 minutes, followed by centrifugation, pelleting, washes with salt wash reagent, centrifugation and dissociation of the collagen–dye complex using an alkali reagent vortexing wash. Absorbance was measured at 595 and 540 nm, respectively, using a plate reader (Chameleon).

### Safranin O staining

Control and sBGN-stimulated cartilage explants were fixed in neutral buffered 10 % formalin for 2 weeks before dehydration in ethanol series, clearing in xylene and embedding in paraffin. Deparaffinised tissue sections were stained with Fast Green dye, rinsed in 1 % acetic acid and stained in 0.1 % Safranin O before evaluation using a Nikon LV-DIA Base microscope (Nikon Instruments, Tokyo, Japan) with a motorized XY staging system (OptiScan III; Prior Scientific, Rockland, MA, USA) connected to a DS-Fi1 digital camera (Nikon Instruments) using NIS-Elements Basic Research analysis (Nikon Instruments). Image analysis was done using ImageJ software (National Institutes of Health, Bethesda, MD, USA).

### Statistical analysis

Differences between groups were tested using the Mann–Whitney *U* test, the Wilcoxon signed-rank test or Student’s *t* test, as appropriate. Effects of covariates were analysed by multiple linear regression. All statistical analysis were performed using IBM SPSS version 21 software (IBM, Armonk, NY, USA). *p* Values <0.05 were considered significant.

## Results

### sBGN and sDCN can be found in synovial fluid obtained from patients with OA or RA

BGN and DCN are key components of the cartilage matrix. We hypothesised that some intact BGN or DCN could be released from matrix into SF in OA or RA and that in soluble form they could act as a proinflammatory stimulus. Indeed, sandwich ELISA disclosed clearly higher sBGN levels in advanced OA (582 ± 307 ng/ml) than the levels found in patients with meniscus tear who had very early OA (14 ± 2 ng/ml) (Table 2). The highest levels of sBGN were observed in SF obtained from patients with RA (1191 ± 482 ng/ml) (Table 2). In contrast to sBCN, the SF levels of sDCN were low, and no differences were observed between early OA (51 ± 4 ng/ml), OA (52 ± 3 ng/ml) and RA: (49 ± 4 ng/ml). Covariates (age, sex and body mass index) tested with multiple linear regression models were not associated with SF sBGN or SF sDCN levels.

**Table 2:** Intact sBGN and sDCN concentration levels in synovial fluid obtained from knee joints of early osteoarthritis, advanced osteoarthritis and rheumatoid arthritis patients

Characteristics	Early OA (*n* = 10)	Advanced OA (*n* = 10)	RA (*n* = 10)
Patient age, yr	63.1 ± 16	64.4 ± 10	55.3 ± 15.4
Males	40 (4)	40 (4)	30 (3)
Body mass index, kg/m^2^	27.2 ± 4.4	27.8 ± 3.5	24.3 ± 4.7
sBGN, ng/ml	14 ± 2	582 ± 307^a^	1191 ± 482^a^
sDCN, ng/ml	51 ± 4	52 ± 3	49 ± 4

*OA* osteoarthritis, *RA* rheumatoid arthritis, *sBGN* soluble biglycan, *sDCN* soluble decorin

Data are presented as mean ± SD or count (%)

^a^ *p* < 0.01 by Student’s *t* test

### sBGN upregulates catabolic factors in OA chondrocytes

**Fig. 1:**
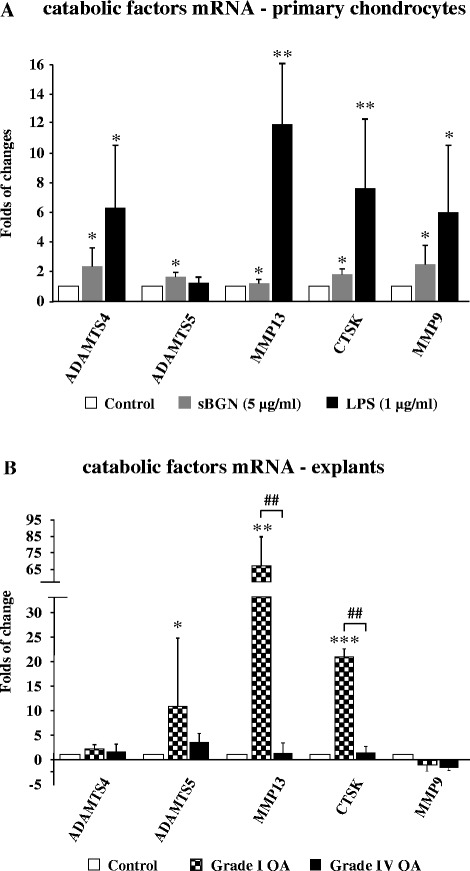
Effect of soluble biglycan (sBGN) on the expression of proteinases associated with matrix remodelling and cartilage degeneration in osteoarthritis (OA). **a** In primary chondrocytes, sBGN increased all proteinases examined. Lipopolysaccharide (LPS) was used as a positive control. **b** In cartilage explants, sBGN increased *ADAMTS5*, *MMP13*, *CTSK* and *MMP9* messenger RNA (mRNA) only in mild grade I OA and not in severe grade IV OA. The results are expressed as fold changes (mean ± SD) relative to TATA box-binding protein (*TBP*) housekeeper and compared with non-stimulated controls. Samples were run as technical duplicates, and each experiment was done using samples obtained from at least six different donors (biological replicates). *p < 0.05, **p < 0.01, ***p < 0.001 vs. non-stimulated controls; ^##^ *p* < 0.01 for pairwise comparisons between mild grade I OA and severe grade IV OA. *ADAMTS* a disintegrin and metalloproteinase with thrombospondin type 1 motif (aggrecanase), *MMP* matrix metalloproteinase, *CTSK* cathepsin K

As high concentrations of intact sBGN were found in the SF of OA and RA patients, we studied whether sBGN could have effects on the cartilage metabolism. First, we studied the effect of sBGN on the expression of catabolic cartilage factors. In primary monolayer chondrocytes, sBGN significantly increased gene expression of *ADAMTS4*, *ADAMTS5*, *MMP13*, *CTSK* and *MMP9* almost as efficiently as LPS (Fig. 1a). Studies of cartilage explant tissues obtained from patients with varying degrees of OA severity revealed that the response to sBGN was dependent on the grade of cartilage degradation. sBGN induced pronounced expression of catabolic factors in grade I OA cartilage, whereas a more modest increase of catabolic factors was observed in highly degenerated grade IV OA cartilage (Fig. 1b).

**Fig. 2:**
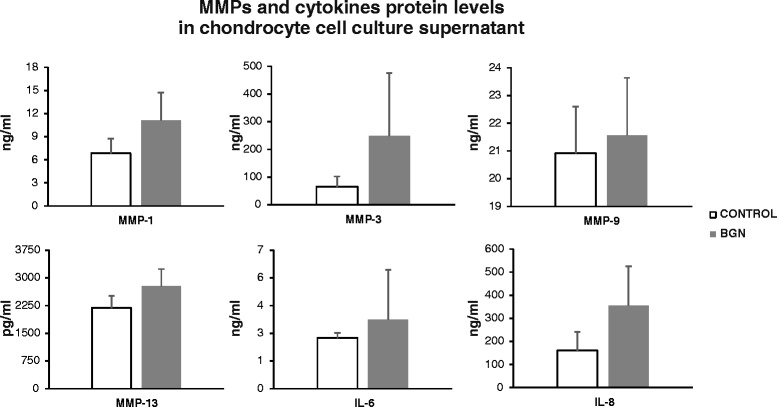
Effect of soluble biglycan (sBGN) on the expression of matrix metalloproteinase (MMP)-1, MMP-3, MMP-9 and MMP-13 as well as interleukin (IL)-6 and IL-8 proteins. sBGN leads to the upregulation chondrocyte-mediated production of major catabolic markers known in osteoarthritis. sBGN-induced concentration levels compared with controls did not reach the statistical significance level <0.05. The results are expressed mean ± SD. Samples were run as technical duplicates, and each experiment was done using samples from three different donors (biological replicates)

To further assess whether the studied gene mRNA transcripts were also translated into proteins, we used Luminex xMAP® technology. In cell culture supernatants, the levels of MMP-1 and MMP-3 were substantially upregulated (1.6- and 3.6-fold, respectively) by sBGN compared with unstimulated controls (Fig. 2). Compared with positive control LPS, the effects of sBGN on protein levels for MMP-1, MMP-3, MMP-9 and MMP-13 were 3 %, 50 %, 82 % and 21 %, respectively, of that of LPS (LPS data not shown).

Levels of proinflammatory cytokines IL-6 and IL-8 were also upregulated by sBGN compared with unstimulated control (1.4- and 2.2-fold, respectively) (Fig. 2). Compared with LPS, the effects of sBGN on cytokine levels for IL-6 and IL-8 were 18 % and 25 %, respectively (LPS data not shown).

### sBGN impairs the synthesis of major cartilage matrix components in OA chondrocytes

**Fig. 3:**
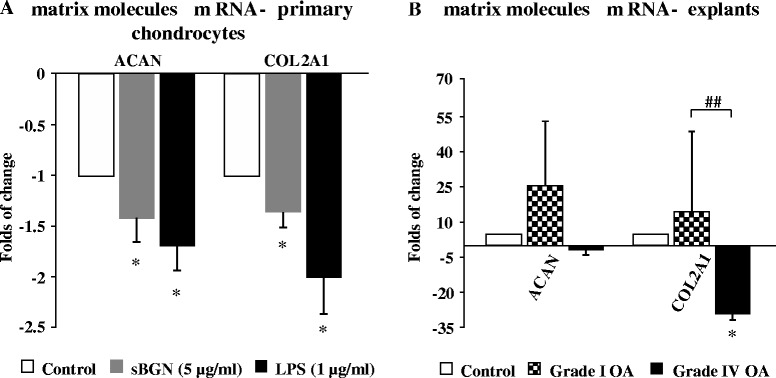
Effects of soluble biglycan (sBGN) on the expression of aggrecan and collagen type II (Col-II). **a** In primary chondrocytes, sBGN decreased aggrecan and Col-II. Lipopolysaccharide (LPS) was used as a positive control. **b** In cartilage explants, sBGN did not affect aggrecan (*ACAN*) or Col-II*(COL2A1)* in grade I osteoarthritis (OA) but decreased Col-II*(COL2A1)* in grade IV OA, with a different response in mild and severe OA. The results are expressed as fold changes (mean ± SD) relative to the TATA box-binding protein (*TBP*) housekeeper and compared with non-stimulated controls. Samples were run as technical duplicates, and each experiment was done using samples obtained from at least six different donors (biological replicates). *p < 0.05 vs. non-stimulated controls; ^##^ *p* < 0.01 for pairwise comparisons between mild grade I OA and severe grade IV OA. mRNA messenger RNA

To determine the effect of sBGN on overall cartilage turnover, we studied the effect of sBGN on the anabolic functions of OA chondrocytes. In primary OA chondrocytes, sBGN significantly inhibited aggrecan and Col-II mRNA expression (Fig. 3a). The studies on cartilage explants revealed that sBGN significantly decreased Col-II mRNA expression in highly degenerated grade IV OA but had no significant effect on the expression in grade I OA cartilage (Fig. 3b).

### sBGN increases proteoglycan and collagen release from cartilage explants

**Fig. 4:**
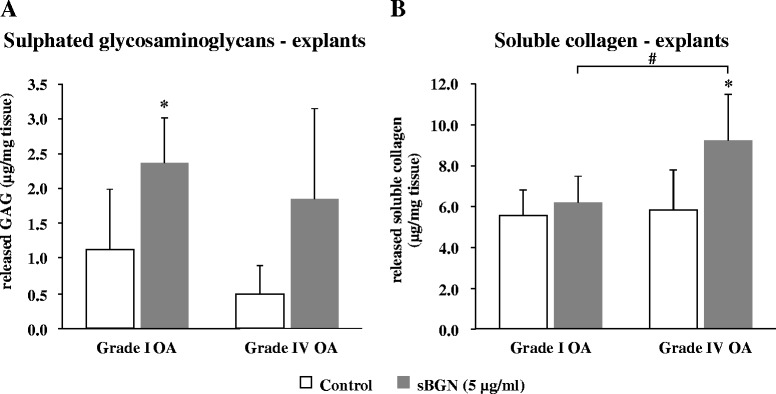
Effects of soluble biglycan (sBGN) on release of glycosaminoglycans (GAGs) and soluble collagen from cartilage explants compared with non-stimulated controls. **a** In grade I osteoarthritis (OA), sBGN increased GAG release. **b** In grade IV OA, sBGN increased collagen release. The results are presented as mean ± SD. Samples were run as technical duplicates, and each experiment was done using samples from at least six different donors (biological replicates). *p < 0.05 vs. non-stimulated controls; ^##^ *p* < 0.01 for pairwise comparisons between mild grade I OA and severe grade IV OA

**Fig. 5:**
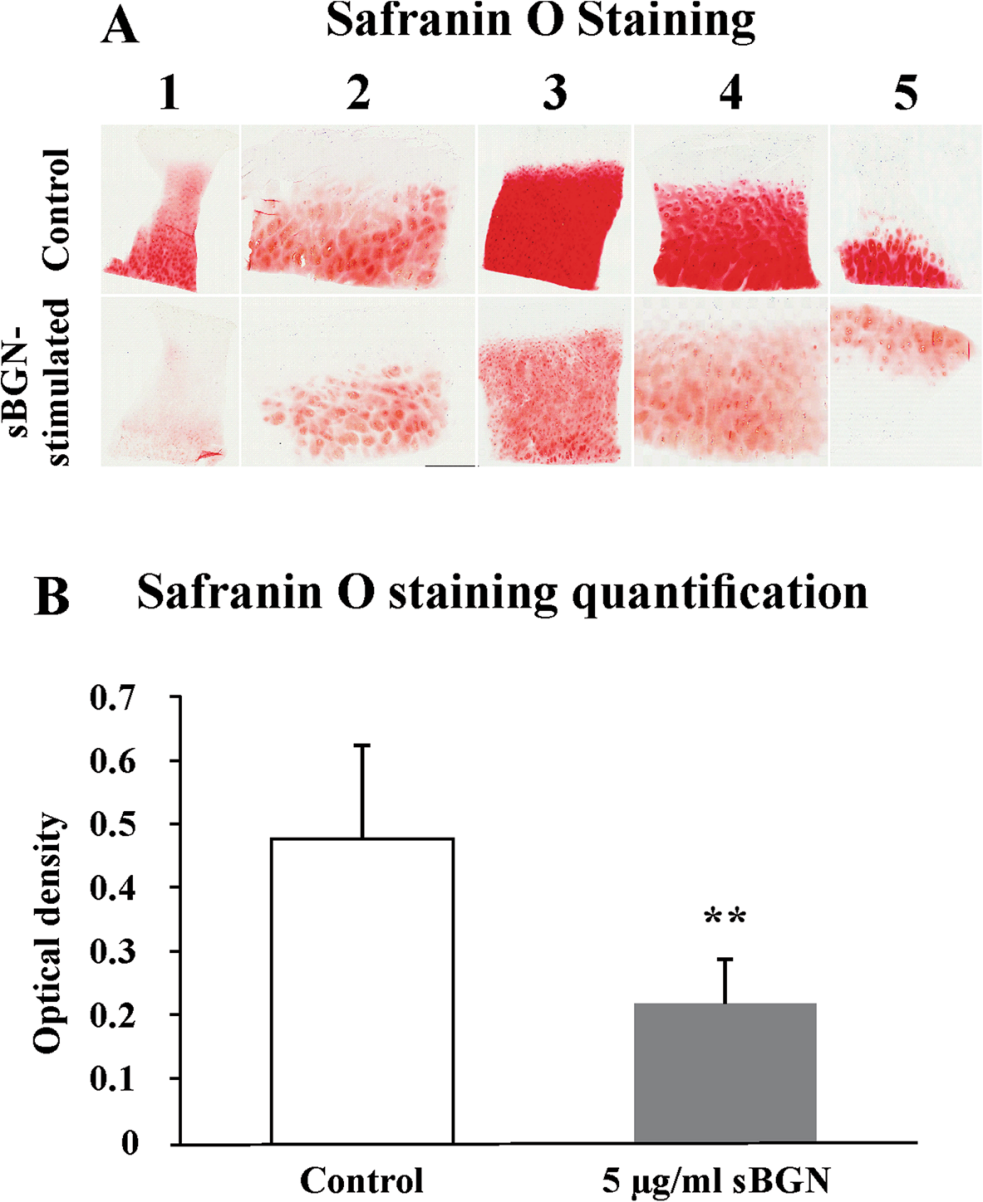
Effects of soluble biglycan (sBGN) on proteoglycan content in osteoarthritis (OA) cartilage explants from five different patients. **a** Safranin O staining of the formalin-fixed, paraffin-embedded tissue sections produced from the OA cartilage explants (4 mm) after 48-h incubation. Note extensive proteoglycan depletion in all samples. Safranin O photomicrographs are panoramic images constructed from several photomicrographs to provide an overall view of cartilage explant extent in one image. Original magnification, ×20. **b** Proteoglycan depletion was quantified by optical density of Safranin O staining using ImageJ image analysis software. The results are presented as mean ± SD. Proteoglycan content was reduced by 50 ± 20 % compared with non-stimulated samples. **p < 0.01

Release of proteoglycans and soluble collagen from cartilage explants into cell culture media was determined to confirm that sBGN can also induce the degradation of OA cartilage by catabolic chondrocytes in situ. sBGN induced prominent release of sGAG in grade I OA explants, whereas sBGN-induced collagen release was observed only in grade IV OA cartilage (Fig. 4). Proteoglycan release was further confirmed by Safranin O staining in sBGN-treated cartilage explants. sBGN decreased the total quantity of proteoglycan in explants by approximately 50 % (Fig. 5).

### sBGN upregulates TLR4 expression in chondrocytes and in cartilage tissue explants

**Fig. 6:**
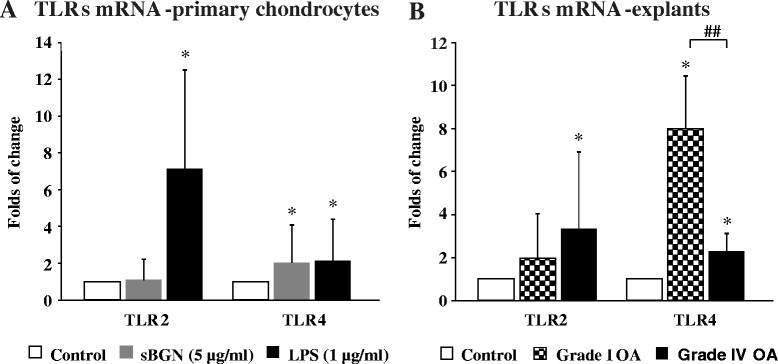
Effect of soluble biglycan (sBGN) on expression of Toll-like receptor 2 (*TLR2*) and Toll-like receptor 4 (*TLR4*) on primary chondrocytes. **a** In primary chondrocytes, sBGN increased *TLR4* (*p* < 0.05) and did it as effectively as lipopolysaccharide (LPS), which was used as a positive control. **b** In cartilage explants, sBGN increased *TLR4*, both in mild grade I (*p* < 0.05) and in severe grade IV (*p* < 0.05) osteoarthritis (OA), but differently in mild and severe OA. The results are expressed as fold changes (mean ± SD) relative to TATA box-binding protein (*TBP*) housekeeper and compared with non-stimulated controls, which corresponds to a fold change of 1. Samples were run as technical duplicates, and each experiment was done using samples obtained from at least six different donors (biological replicates). *p < 0.05 vs. non-stimulated controls; ^##^ *p* < 0.01 for pairwise comparisons between mild grade I OA and severe grade IV OA

To study the mechanism of the effects of sBGN on cartilage metabolism, the effects of sBGN on TLR expression and function were first studied. Expression of *TLR2* and *TLR4* mRNA was observed in primary monolayer chondrocytes and cartilage explants of all patients with OA. In accordance with earlier studies, basal *TLR2/TLR4* levels were slightly (1.3-fold) higher in grade IV OA than in grade I OA [30]. In primary monolayer chondrocyte cultures, sBGN significantly increased *TLR4* expression but did not have any significant effect on *TLR2* mRNA expression (Fig. 6a).

**Fig. 7:**
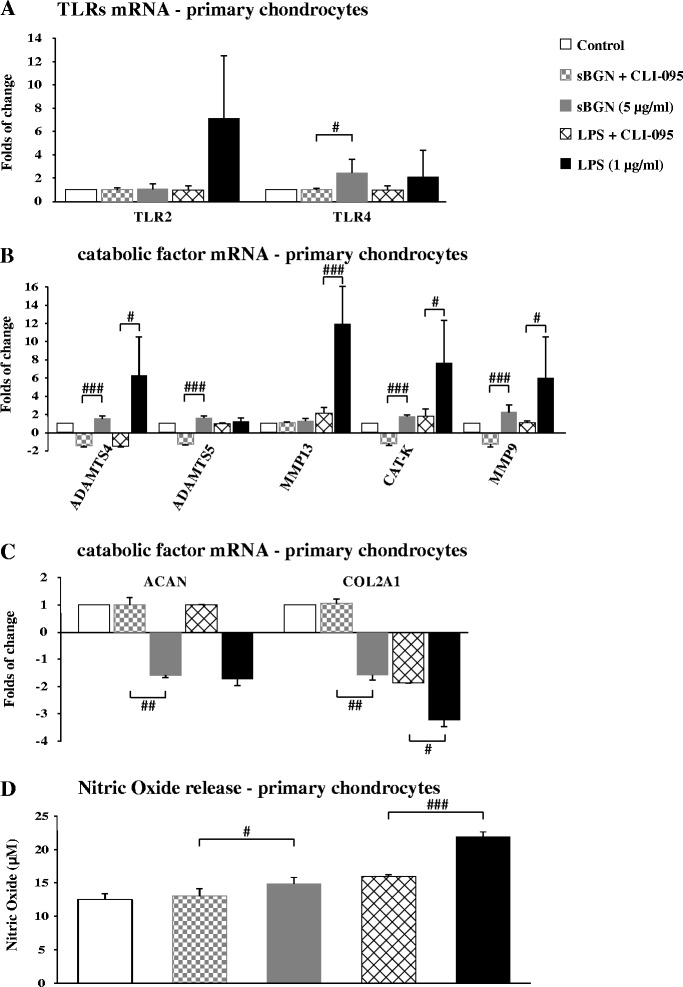
CLI-095 abrogates soluble biglycan (sBGN)– and lipopolysaccharide (LPS)–induced Toll-like receptor 4 (TLR4) signalling with CLI-095 in primary chondrocytes. **a** Pre-incubation with CLI-095 before sBGN and LPS stimulation abrogated the increase in *TLR4* messenger RNA (mRNA) expression. **b** A minor increase in catabolic factor mRNA expression occurred when primary chondrocytes were treated with CLI-095 before sBGN and LPS stimulation. **c** Blocking TLR4 signalling with CLI-095 inhibited the sBGN-induced upregulation of aggrecan (*ACAN* or *ADAMTS*) and collagen type II (*COL2A1*) before stimulation. **d** Production of nitric oxide is reduced by CLI-095 incubation before sBGN or LPS stimulation. All in vitro experiments were technically repeated at least four times with primary chondrocytes (obtained from one biological sample). Differences between mean values were compared using Student’s *t* test, assuming normal distribution where four replicates were used. ^#^ *p* < 0.05, ^##^ *p* < 0.01, ^###^ *p* < 0.001 for pairwise comparisons between CLI-095 pre-incubated and non-CLI-095 pre-incubated sBGN or LPS stimulation. *CTSK* cathepsin K, *MMP* matrix metalloproteinase

Studies of cartilage explants revealed that the TLR response to sBGN is dependent on the grade of cartilage degradation. In grade I OA, the sBGN-induced increase of *TLR4* mRNA expression was 352 % than that observed in grade IV OA cartilage (Fig. 6b). In contrast, there was no difference in sBGN-induced *TLR2* mRNA expression between grade I and grade IV OA (Fig. 6b). Blocking TLR4 by using a low molecular weight inhibitor of TLR4 signalling (CLI095) abrogated the sBGN-induced increase of *TLR4* mRNA expression (Fig. 7a).

### Catabolic effect of sBGN in articular chondrocytes is mediated via TLR4

**Fig. 8:**
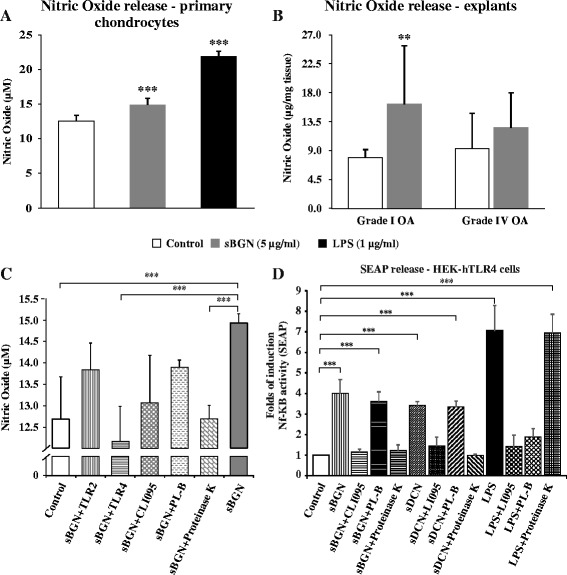
Effects of soluble biglycan (sBGN) on HEK-hTLR4 secreted embryonic alkaline phosphatase (SEAP) production and chondrocyte production of nitric oxide (NO) compared with control groups. **a** In primary chondrocytes, sBGN increased NO. Lipopolysaccharide (LPS) was used as a positive control. **b** In cartilage explants, sBGN increased NO only in grade I osteoarthritis (OA) without a difference between mild and severe OA. **c** Addition of polymyxin B had no effect on the sBGN-induced NO production, while proteinase K restored NO production to levels seen in controls, indicating that there was no endotoxin contamination of sBGN. Addition of Toll-like receptor 2 (TLR2)–neutralising antibody caused no significant reduction of sBGN-mediated NO production, while TLR4 neutralisation impaired NO production. Samples were run as technical duplicates, and each experiment was done using samples from at least six different donors (biological replicates). **d** Effect of sBGN, soluble decorin (sDCN) and LPS on TLR4 activity. An SEAP assay was used to determine nuclear factor (NF)-κB activity following sBGN, sDCN or LPS stimulation of HEK-hTLR4. The results are from three independent experiments. All results are expressed as mean ± SD. **p < 0.01, ***p < 0.001 vs. non-stimulated controls

Inducible nitric oxide synthase (iNOS) is activated via TLR2/TLR4/NF-κB pathway activation. To further evaluate the role of TLRs in sBGN-induced signalling, we studied the effect of sBGN on NO release from primary OA chondrocytes. In primary OA chondrocyte cultures, sBGN significantly increased NO production (Fig. 8a). In cartilage explant tissues, sBGN increased NO production slightly more in grade I OA than in grade IV OA (Fig. 8b). Blocking of TLR4 signalling with TLR4-neutralising antibody or synthetic inhibitor CLI-095 significantly reduced NO production to levels observed in non-stimulated controls (Fig. 8c), suggesting that the effect of sBGN is mediated via TLR4. To exclude the effect of contaminating LPS, polymyxin B was added, which had no effect on the sBGN-induced production of NO. In contrast, proteinase K reduced NO production to levels of non-stimulated controls, further supporting the finding that the observed effect of sBGN is not caused by LPS contamination. Moreover, blocking of TLR4 signalling abrogated the increase in mRNA expression of the catabolic factors induced by both sBGN and LPS while also restoring to normal the levels of the mRNA expression of the key anabolic factors *ACAN* and *COL2A1* (Fig. 7b and c). In contrast to TLR4, TLR2-neutralising antibody had no significant effect on NO production. Together, these above suggest that the effect of sBGN on cartilage metabolism is mediated via activation of TLR4 and that TLR2 has either no or only a minor role.

To confirm that sBGN signalling is mediated through TLR4/NF-κB pathway, we studied the activation of NF-κB after stimulation with sBGN. This was done using engineered HEK-hTLR4 cells, which can be stimulated only through activation of the TLR4 receptors. These cells have been stably transfected with SEAP plasmid containing NF-κB response elements, and the activation of NF-κB can be monitored by measuring activity of SEAP. Stimulation of HEK-hTLR4 cells with sBGN caused a substantial increase of SEAP, while inhibition of TLR4 signalling with TLR4 inhibitor CLI-095 prevented the increase of SEAP levels. sDCN and LPS caused a similar increase of SEAP levels. Proteinase K abrogated SEAP upregulation by sBGN and sDCN, while polymyxin B did not alter the response, thus ensuring that the studied effect was not caused by endotoxin contamination (Fig. 8d).

## Discussion

BGN and DCN are two major non-collagenous SLRP products of chondrocytes which are deposited into articular cartilage matrix [13]. In this study, we show, for the first time to our knowledge, that intact sBGN is present in SF obtained from patients with meniscus tear lesions and early OA, advanced OA or RA, with the highest concentrations found in advanced OA and RA. DCN could also be found in SF, but, in contrast to BGN, only low levels were detected and no differences between early OA, advanced OA and RA were observed. Thus, matrix-embedded chondrocytes as well as other cells in joints are exposed to fluid phase sBGN and sDCN. Intact sBGN and sDCN, but not their fragments, have been shown to engage proinflammatory responses [31, 32]. Therefore, while BGN fragments are known to be released into the SF of patients with OA, their presence is merely a reflection of disease activity. Hence, they have been proposed to be solely a diagnostic biomarker. The observed increased levels of intact sBGN are also in line with the observed upregulation of mRNA and protein levels of sBGN in human OA cartilage tissue and also in cartilage obtained using a sheep meniscectomy animal model of OA. Although DCN mRNA and protein levels have been shown to increase in OA, this was not reflected in the level of DCN in SF [22, 25, 33]. Interestingly, SF of OA and RA patients has been shown to contain immunoglobulin G autoantibodies against BGN, DCN and several other cartilage matrix molecules, suggesting their release from matrix and subsequent local loss of immunological tolerance [34]. Immune complex formation leading to complement and Fcγ receptor activation plays a role in RA, as well as in OA according to recent studies [35–37]. The present finding of intact sBGN in SF, together with the recognition of sBGN as an endogenous damage-associated molecular pattern-type ligand for TLR2/TLR4 and for P2X_7/4_, provides a third mechanism of relevance for autoinflammation, co-stimulation and autoimmunity.

Chondrocytes of OA cartilage are equipped with a full palette of TLRs, including TLR2 and TLR4, and the proportion of TLR^+^ chondrocytes increases with progression of OA [38]. The results of the present study show that sBGN regulates TLR4 expression to an extent similar to that of LPS, which is a known TLR2/TLR4 ligand. The effects of sBGN were not caused by LPS contamination, as the result of the endotoxin test was negative and polymyxin B did not have any effect on the results in HEK-hTLR4 cells or in primary chondrocytes. Thus, sBGN can be conceived of as an endogenous damage-associated molecular pattern, a cartilage-derived LPS mimic. The mechanism of action of sBGN and DCN is mediated through activation of TLR4 and the NF-κB pathway. This is supported by several lines of evidence. In HEK-hTLR4, which can be activated only through TLR4, sBGN and sDCN induced the secretion of AP, a reporter of NF-κB activation. This increase was significantly inhibited by CLI-095, an inhibitor of TLR4 signalling. Furthermore, in primary chondrocytes, sBGN induced production of NO, which was almost completely impaired by CLI-095, an inhibitor of TLR4 signalling, and also by TLR4-neutralising antibody. In contrast, addition of TLR2 antibody had no significant effect on sBGN-induced NO production (Fig. 8). The signalling of TLR4 has been shown to occur via NF-κB, which in turn is the principal inducer of iNOS [39, 40]. Furthermore, NO has been implicated as an important pro-inflammatory mediator of inflammation in OA [41, 42]. Thus, the sBGN induction of secreted AP in HEK-hTLR4 cells and the NO production in chondrocytes suggests that sBGN can induce a chondrocyte-mediated inflammatory response in cartilage.

sBGN has been shown in earlier in vitro experiments to exert dose-dependent effects at least up to 80 μg/ml concentration on peritoneal macrophages [43]. Although sBGN concentrations used in vitro are somewhat higher than those now measured in SF, they are probably pathophysiologically relevant. Given the fact that BGN is a pericellular matrix proteoglycan, local concentrations in the vicinity of chondrocytes can be expected to be higher than in SF [44]. Moreover, lack of biomechanically cyclic compression of the cartilage explants causes a drop in interstitial fluid pressure, and therefore higher doses of sBGN are needed to ensure outside-in access. Nevertheless, current in vitro techniques cannot mimic in vivo conditions, and therefore the next step would be to replicate the findings of the present study in an OA animal model.

Interaction of sBGN with TLR4 (and possibly to a lesser extent the other sBGN receptor, P2X_7/4_) led to increased production of aggrecanases and collagenolytic enzymes at the mRNA and protein levels [45]. After cleavage across the collagen triple helix, fragments undergo spontaneous helix-to-coil transition to gelatin at body temperature. Thus, the sBGN-induced proteoglycanases (ADAMTS-4/5), collagenases (MMP-13, cat K) and gelatinases (MMP-9) are able to degrade all the major components of the hyaline articular cartilage. The observed elevated production of IL-6 and IL-8 levels induced by sBGN in chondrocytes may also lead to increase recruitment and influx of inflammatory cells such as neutrophils and macrophages [46, 47]. Together the observed levels of MMPs and cytokines add further molecular stress to the OA joint and might create positive feedback loop of inflammation and cartilage degradation.

Soluble BGN induced expression of proteinases clearly more effectively in grade I than in grade IV OA. Grade IV OA represents an advanced stage of the disease and the low response of grade IV OA to sBGN could represent *functio laesa* (i.e., function disturbed as a result of inflammation and loss of cartilage). Interestingly *BGN* mRNA is more upregulated in advanced OA compared with early stages suggesting in fact that desensitisation to BGN in grade IV OA explants may have occurred due to long term exposure at the pericellular lacunae or by an attempt of the cartilage to compensate the proteoglycan loss [22]. In addition, *TLR4* upregulation by sBGN was substantially higher in grade I OA vs grade IV OA. Thus as expected TLR4-mediated NF-κB pathway products (i.e. proteinases and aggrecanases) were also proportionally upregulated. Likewise NO, a product of iNOS which is regulated by NF-κB pathway, was also increased in grade I OA vs. grade IV OA.

In primary chondrocytes sBGN inhibited synthesis of the collagen (fibre network) and aggrecans (ground substance). In relatively well preserved grade I OA explants sBGN had a slight stimulatory effects on matrix synthesis, whereas in more advanced grade IV OA Col-II synthesis decreased significantly. Such an imbalance between degradation and synthesis results in a net loss of cartilage [48, 49].

Due to their potential destructive properties, proteolytic enzymes are tightly regulated. ADAMTS-4/ADAMTS-5 and MMPs are regulated on the transcriptional level (e.g., by sBGN), as shown in the present study. They are synthesised as latent pro-enzymes, which for activation require proteolytic removal of the activation (pro)peptide [50, 51]. The MMPs are regulated by tissue inhibitors of metalloproteinases. Therefore, the most direct way to demonstrate sBGN-induced proteolysis of cartilage is to demonstrate a release of proteoglycan and collagen degradation products. This was done by using GAG and collagen-binding dyes and light absorption. Proteoglycan release was statistically significant only in grade I OA, as expected given the substantially higher catabolic response by grade I OA chondrocytes. In contrast, collagen release was significant only in type IV OA, perhaps because collagen becomes accessible for collagenolytic enzymes only after proteoglycan depletion [52]. Release of proteoglycans was confirmed by staining, which revealed their depletion from cartilage matrix in partly overlapping samples.

## Conclusions

We demonstrate that intact sBGN is present in knee SF of patients with advanced knee OA or RA, whereas only low amounts of sDCN could be detected. sBGN upregulates *TLR4* expression in chondrocytes, increases the expression and concentration levels of catabolic factors and decreases the expression of anabolic factors, resulting in net loss of cartilage. The mechanism of action of sBGN in chondrocytes is mediated mainly through activation of TLR4. These results support the importance TLR4 signalling in OA pathomechanisms, and thus TLR4 may be a potential therapeutic target in inflammation- and catabolism-mediated cartilage degenerative disorders. Importantly, our findings strongly support the role of sBGN, a major ECM protein, as a potential biomarker, therapeutic target and contributor in the catabolic events that occur during progression of OA.

## Abbreviations

ACAN: aggrecan
ADAMTS: aggrecanase
AP: alkaline phosphatase
Col-II: collagen type II
CTSK: cathepsin K
DMEM: Dulbecco’s modified Eagle’s medium
ECM: extracellular matrix
ELISA: enzyme-linked immunosorbent assay
FCS: foetal calf serum
GAPDH: glyceraldehyde 3-phosphate dehydrogenase
IL: interleukin
iNOS: inducible nitric oxide synthase
LPS: lipopolysaccharide
MMP: matrix metalloproteinase
mRNA: messenger RNA
NF-κB: nuclear factor-κB
NO: nitric oxide
OA: osteoarthritis
PBS: phosphate-buffered saline
RA: rheumatoid arthritis
sBGN: soluble biglycan
sDCN: soluble decorin
SEAP: secreted embryonic alkaline phosphatase
SF: synovial fluid
sGAG: sulphated glycosaminoglycan
SLRP: small leucine-rich proteoglycan
TBP: TATA box-binding protein
TKA: total knee arthroplasty
TLR: Toll-like receptor
